# Low-level laser effect in patients with neurosensory impairment of mandibular nerve after sagittal split ramus osteotomy. Randomized clinical trial, controlled by placebo

**DOI:** 10.4317/medoral.19626

**Published:** 2014-03-08

**Authors:** Alberto Führer-Valdivia, Alfredo Noguera-Pantoja, Valeria Ramírez-Lobos, Pedro Solé-Ventura

**Affiliations:** 1 Dental Surgeon: Universidad de los Andes, Chile; 2 Dental Surgeon, Public Health Magister, Universidad de los Andes, Chile; 3 Maxillofacial Surgeon, Universidad de los Andes, Chile

## Abstract

Objectives: Evaluate the effect on the application of low level laser therapy, in patients that have been previously intervened with a sagittal ramus split osteotomy and present neurosensory impairment due to this surgery, compared with placebo. Study Design: This preliminary study is a randomized clinical trial, with an experimental group (n=17) which received laser light and a control group (n=14), placebo. All participants received laser applications, divided after surgery in days 1, 2, 3, 5, 10, 14, 21 and 28. Neurosensory impairment was evaluated clinically with 5 tests; visual analog scale (VAS) for pain and sensitivity, directional and 2 point discrimination, thermal discrimination, each one of them performed before and after surgery on day 1, and 1, 2 and 6 months. Participants and results evaluator were blinded to intervention. Variables were described with absolute frequencies, percentages and medians. Ordinal and dichotomous variables were compared with Mann Whitney’s and Fisher’s test respectively. Results: Results demonstrate clinical improvement in time, as well as in magnitude of neurosensory return for laser group; VAS for sensitivity reached 5 (normal), 10 participants recovered initial values for 2 point discrimination (62,5%) and 87,5% recovered directional discrimination at 6 months after surgery. General VAS for sensitivity showed 68,75% for laser group, compared with placebo 21,43% (p-value = (0.0095). Left side sensitivity (VAS) showed 3.25 and 4 medians for placebo and laser at 2 months, respectively (p-value = (0.004). Conclusions: Low-level laser therapy was beneficial for this group of patients on recovery of neurosensory impairment of mandibular nerve, compared to a placebo.

** Key words:**Laser Therapy, low-level, LLLP, osteotomy, sagittal split ramus, paresthesia, mandibular nerve.

## Introduction

Bilateral sagittal split osteotomy (BSSO) procedures, generates by itself, complications such as mandibular nerve damage, classified as neuropraxia, axonotmesis and in more severe cases neurotmesis (1,2). This damage involves Aα y Aβ fibers responsible for mechanoception (touch) and Aδ y C fibers, responsible for pain and temperature. This phenomenon has clinical manifestations like disturbances on: tactile directional discrimination, 2-point discrimination, pain and thermoalgesic discrimination. The reported incidence of these alterations varies between 85-87% (1,3). Colella’s systematic review reveals neurosensory impairment after surgery with objective and subjective neurosensory tests; 63,3% and 83% respectively at day 7 postoperative (4). According to severity, a retrospective study evaluated the incidence of post, intra and preoperatory complications due to orthognatic surgery. Results reveal that the most frequent complication was neurosensory impairment in mandibular nerve innervation area, being mild in 32% of patients and severe in 3% of them (5).

Mandibular nerve damage, may occur at mandibular foramen, along its path through the canal, or in relation to mental foramen. Despite this, symptoms referred to nerve injury varies on different degrees of lower lip and chin paresthesia, mental nerve distribution area (1). Neurovascular damage associated to BSSO, correlate a variety of factors that should be considered to comprehend the presence of these complications and its posterior recovery, including advanced age of patients, magnitude and direction of mandibular movement, unfavorable osteotomy, mandibular nerve manipulation, intraoperative excessive bleeding, associated surgery (genioplasty), simultaneous third molar removal, use of rigid or intermaxillary fixation, local anesthetic use, experienced surgeon (1,6,7).

Neurons try to repair damage in sections or injuries of peripherical nerve fibers. This leads to repair and regeneration process within multiple structural and metabolic phenomena, reestablishing their functions. On the other hand, when a traumatic phenomenon destroys central nervous system cells, these cannot be replaced due to their inability to proliferate, determining permanent nerve damage. Spontaneous recovery of incomplete nerve lesions is often unsatisfactory. Normal results of these non-treated lesions are axon degeneration. There are cases where recovery can happen, but it seems to be in a partial and paused way. Therefore, the numerous attempts trying to improve, or accelerate treatment research (8-10) are understandable. Low-level laser therapy as a treatment, seeks to accelerate recovery, decrease postoperative pain and restore normal function of the injured nerve, among other functions. There are studies which use low level laser therapy in mandibular nerve paresthesia, showing an increase in time and magnitude of neurosensory recovery (1,11,12).

The objective of this study was to evaluate the effect on the application of a low-level laser therapy, in patients that have been previously intervened with a BSSO and present neurosensory impairment due to the surgery, compared to placebo.

## Material and Methods

This study is a randomized clinical trial with 2 parallel groups (1:1), defined as experimental and control groups. The population involved is individuals that were treated surgically after being diagnosed with dentomaxillar desarmonies and previous orthodoncist management. All participants were intervened with a BSSO using a short reciprocating blade (REF 5100-37, cut edge 14,5 mm, thickness 0,38 mm) for use with TPS reciprocating saw (Stryker CORE, Kalamazoo, Michigan State, USA) under local and general anesthesia. Procedures were located in 3 private hospitals in Santiago, Chile. In terms of eligibility criteria for participants, there were no restrictions about age, gender or patients skeletal class. Pharmacologically, antibiotics, non-steroidal anti-inflammatory (NSAIDs) and corticoids were given to patients. Inclusion criteria were: all participants that undergo a BSSO by the same maxillofacial surgeon and present neuropraxia or axonotmesis due to BSSO with clinical manifestations of either paresthesia, dysesthesia, hypoesthesia or complete anesthesia, patients without complete or partial mandibular nerve section observed by surgeons during surgery. Exclusion criteria included patients with: head and neck tumors, infections, non diagnosed injuries, treatment based on endogenous photo sensible drugs (tetracycline, griseofulvins, sulfonamides, furocumarin) or exogenous (retinoic or glycolic acid).

Sample size consisted of 33 patients (n=33) that voluntarily agreed to participate in the study. Each experimental group participant received 8 low level laser applications, distributed three of them during their hospitalization (days 1, 2 and 3 postoperative) and the other five left with postoperative surgery evaluations at days 5, 10, 14, 21 and 28 postoperative, as well as clinical neurosensory tests (days 1 and 1, 2 and 6 months). The control group received the same laser applications and clinical neurosensory tests with laser light turned off, acting as a placebo. Laser and neurosensitive evaluations ( Table 1) were taken from literature (1,3,5-7,11-14). Local Ethics Committee of Universidad de los Andes approved protocol and interventions of this investigation. All patients gave informed consent for participation in this study.

**Table 1:**
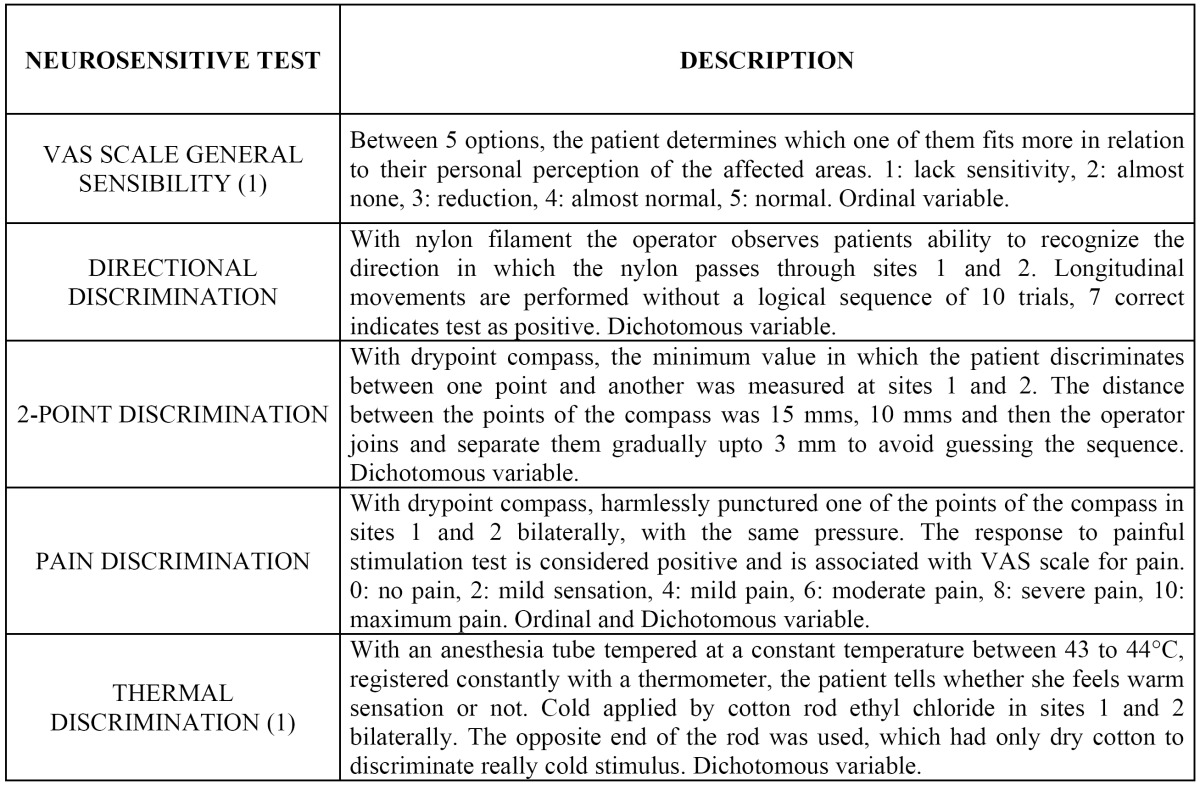
Description of neurosensory evaluation.

- Low level laser application

Gallium – aluminum – arsenide - diode (GaAlAs) low-level laser was used for each session (Flash Lase III, DMC equipment, Sao Paulo, Brazil; 810+/-20nm, 100mW, spot area 0,283 cm2). This laser equipment was previously calibrated for its use. Laser operators and participants used specific protection glasses. Additionally, participants were bandaged. Two laser operators were trained and calibrated for its use. Intraoral infrared laser light application sites for all participants were both left and right sides: mandibular and mental foramen and osteotomy site (buccal side in relation to mandibular second molar). Laser application technique consisted in punctual way. Each site (3 intraoral) received 90 seconds of laser application (32J/cm2, 9J per site) (1,12,11). Intermaxillary orthodontic elastics were taken out during laser application. Placebo group received the same applications of laser group, but laser light was turned on and off immediately for their application so that the laser timer noise help confusing the patient whether he or she were receiving laser light.

- Neurosensory evaluation 

Neurosensory evaluations were made: basal (previous BSSO), 24 hours after surgery (neurosensory impairment indicator), 1, 2 and 6 months postoperative. Evaluations were performed on each patient in a dark, quiet and comfortable room with patients eyes closed. Procedures were explained and demonstrated to all participants before the execution, using patient’s hands as a control site. Each evaluation consisted of five individual tests ( Table 1), each of them performed by the same operator in the same sequence, but not in the same order within each test. This neurosensory evaluation is the result of other neurosensory tests gathered in literature (1,3,5,6,12,11,13-16). Between each test, patients were asked to take off eye bandages, for the next test explanation. Two anatomic sites were determined for each left and right sides: site 1 was lower lip and site 2 was 20 mm below oral commissure, drawing an imaginary vertical line from this point (in relation to the mentolabial sulcus).

The independent variable was the exposure or non-exposure to laser light. Dependent variables were neurosensation measured by dichotomous tests (tactile directional discrimination, 2-point, pain and thermoalgesic discrimination) and ordinal tests (VAS for pain and sensitivity). Other factors were correlated with potential increase in neurosensory impairment after BSSO including age, skeletal class, direction and magnitude of mandibular movement, intraoperative mandibular nerve manipulation, associated mandibular surgery (genioplasty), simultaneous third molar removal, fixation methods and mandibular nerve accident.

Sample size was determined arbitrarily for this study, according to BSSO realized by the surgeon between June 2011 -September 2012. Participants were randomly assigned following simple randomization procedures before treatment, in experimental and control group using Stata 11: Data Analysis and Statistical Software (Stata Corp, College Station, TX). Centralized assignment with a third person that communicates via email, giving participants allocation was used. Neurosensory examiner and patients were blinded to intervention until 6 months after surgery.

Medians and interquartile range (IQR) were used to describe ordinal variables and relative and absolute frequencies for dichotomous variables. The group’s comparison for ordinal and dichotomous variable was made with Mann Whitney’s and Fisher’s test respectively.

## Results

**Figure 1:**
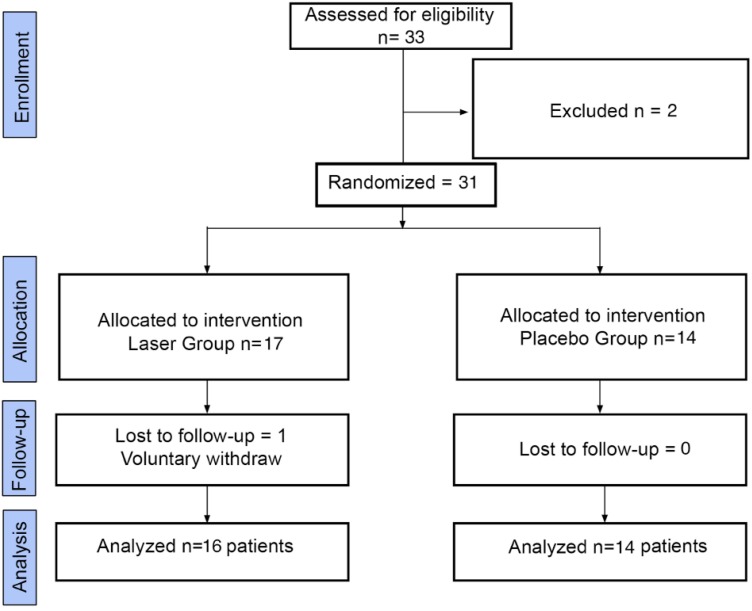
Flow diagram of participants.

Participants flow is showed in figure 1. Two patients right mandibular nerve were divided, hence results obtained on that side of those patients was excluded from results. Both patients received a neurorrhaphy. One participant decided voluntarily to withdraw from the study. Even though all participants were blinded to intervention, some patients of the laser group felt a slight tingling after laser applications.

**Figure 2:**
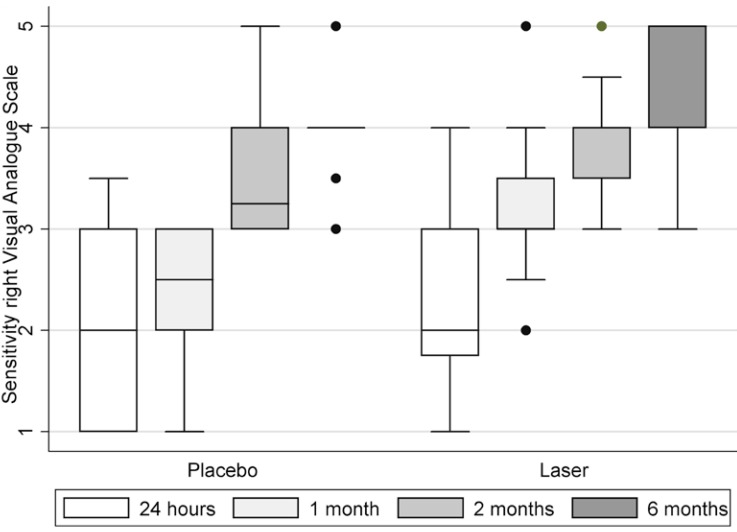
Sensitivity description right by Visual Analogue Scale (VAS) for 24 hrs, 1, 2 and 6 months.

**Figure 3:**
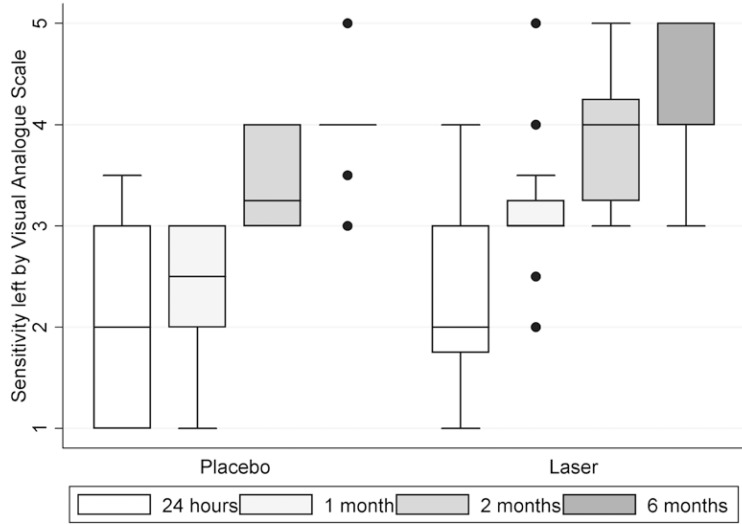
Sensitivity description left by Visual Analogue Scale (VAS) for 24 hrs, 1, 2 and 6 months.

 Table 2 shows demographic and clinical characteristics of participants. General VAS for sensitivity showed 68,75% of recovery for laser group, compared with placebo 21,43% (*p*-value = (0.0095) at 6 months after surgery. Both right and left sides showed improvement in time with VAS sensitivity test. Between placebo and laser groups, VAS sensibility scale for right side at 1 month showed medians of 2,5 and 3 respectively (*p*-value (0.0043) (Fig. 2); For left side, medians at 1 month were 2,5 and 3 respectively (*p*-value (0.0055) (Fig. 3) and at 2 months were 3,25 and 4 respectively (p-value (0.004) (Fig. 3). 2-point discrimination test showed that 9 patients, all of them from the laser group, recover normal sensitivity 2 months after surgery (*p*-value (0.0521). In the same test, at 6 months after surgery, 62,5% of laser group patients completely recovered (*p*-value (0.0631). For directional, algesic and thermal discrimination, results were not favorable for any of the two groups.

**Table 2:**
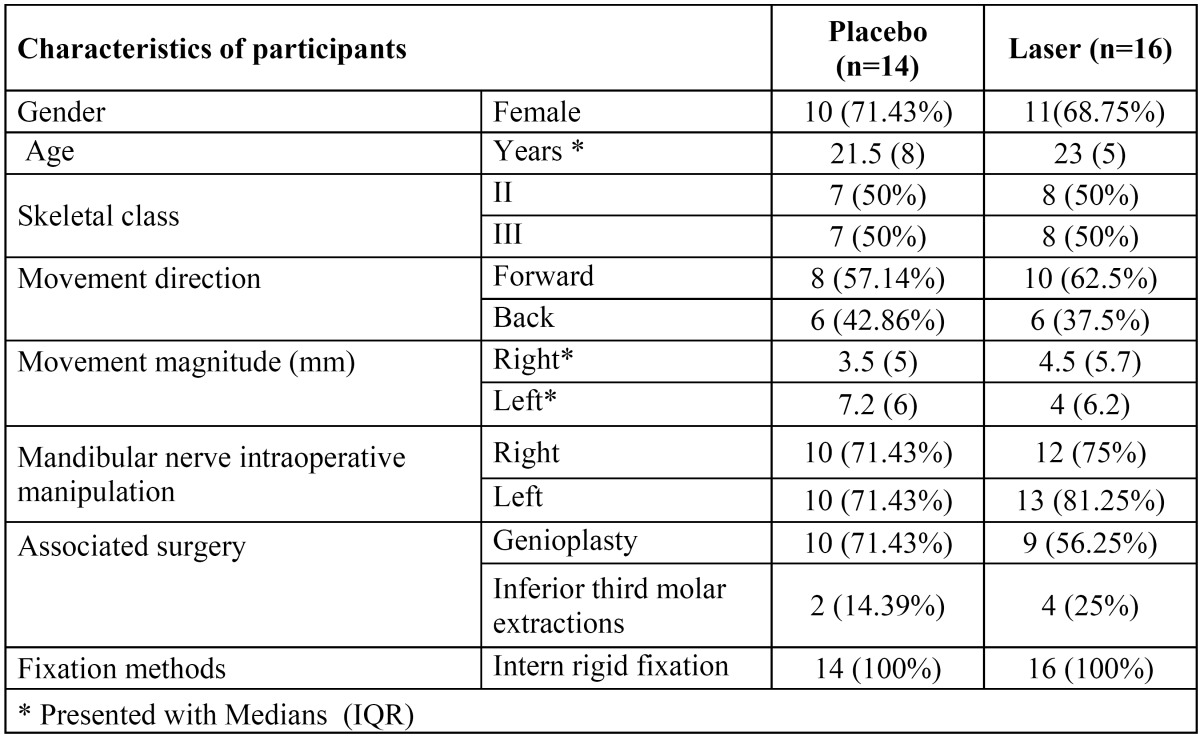
Demographic and clinical characteristics of placebo and laser groups.

There was no adverse effect reported on the application of low-level laser therapy on participants in this study.

## Discussion

BSSO procedures, generates by itself, complications such as mandibular nerve damage, classified as neuropraxia, axonotmesis and in more severe cases neurotmesis (1,2). Results show clinical improvement in time, as well as in magnitude of neurosensory return for the laser group. Lower lip and chin skin indeed were the sites of major neurosensory affection, which was found by study participants and neurosensory test operator. The literature supports this statement, determining them as the sites of major neurosensory affection because of a BSSO (1,13,17,18).

All study participants were able to define quite precisely, the area of the skin that had an abnormal or maintained sensation in relation to the sensitivity of the affected area. These, relate tingling and itching, usually in controls at months 1 and 2 postoperative. Added to these sensations, they relate discomfort associated with the cold environment. Facing the neurosensory assessment, some patients experienced anxiety to respond correctly to stimuli presented to them and often tried, in different evaluation times, to decode the order within each test and answer inconsistently against the stimuli applied to them.

We observed the beneficial effect of low level laser therapy based on GaAsAl on the restoration of mandibular nerve neurosensory function, like similar studies (Miloro M. Repasky 2000; Khullar S. 1996; T. Ozen 2006) (1,11,12) that evaluated sensory changes after BSSO and mandibular third molar extraction and the application of a therapeutic laser, with different patterns of laser applications and neuropraxia evaluations (1,13,17,18).

Study limitations are evidenced, which are that each participant was intervened with surgery, which was planned according to the particular needs of each individual clinical case. Therefore, it is important to consider the individual characteristics of each patient’s anatomy, as well as intraoperative events of each surgery performed and patients postoperative evolution (1,6,7).

Low level laser application is an alternative treatment to restore normal neurosensory function in sites with some degree of alteration and therefore, the response to this therapy is conditioned by all the factors previously mentioned, especially the neurosensory damage degree. The presence of the opening of the ramus in BSSO, determines that disturbances generated manifest as an inherent complication of the technique, being one of the principle causes of neurovascular damage. Along with this, neurosensory disorders could occur due to strain or nerve compression generated by the advance or mandibular retrusion. All cases performed some degree of nerve injury in relation to the mandibular nerve, mainly because it is involved in the mandibular fracture separation. This triggers different degrees of neurosensory impairment of the lower lip and chin. Participants, as expected, had varying degrees of nerve damage. The neurapraxia clinically manifested as sensitivity alterations, anesthesia, hypoesthesia, dysesthesia or paresthesia, burning pains sensations, tactile and thermoalgesic alterations (1,2).

This study was conducted with men and women systemically healthy, of various ages, II and III skeletal class. This allows for a reproduction in a wide spectrum population of patients undergoing orthognathic surgery.

In relation to laser protocol therapy, there are several alternative schema laser applications. Most agree, that there should be a minimum of ten sessions, ideally three times per week, where the low level laser can be used intra and extra orally, using the light directly on the affected area as described above (1,11,19).

General sensitivity recovery results in this study were similar for both groups during the first 2 months postoperatory. However, normal recovery was reached by a major number of patients from experimental group, suggesting a beneficial effect of low level laser therapy in neurosensory impairment of mandibular nerve during the first 6 months after BSSO surgery. Further clinical trials are necessary to support laser protocol applied in this study and the effect on nerve recovery after BSSO.

Biological mechanism of low level laser therapy for neurosensory recovery has not been fully clarified. Current theories suggest that laser produces effects on cellular metabolic levels, resulting in the stimulation of light sensitive fibers or enzymes (rhodopsin kinase) of damaged axons. Tissue cells so as nerve fibers, absorb stimulation delivered by the infrared laser light (790 and 830 nm), generating: ATP mitochondrial, production of certain proteins, activation of cellular enzymatic processes and the increase of intracellular calcium (1,9,11,19,20). Other theories suggest that injured axons and Schwann cells due to laser application, produce a self-regulation and thereby increase the regeneration of injured axons through the production of certain neurotrophic factors (20).

A possible neuroprotective mechanism has been described for laser light through the removal of nitric oxide activity, neurotoxicity generating agent. Laser application has also showed the reduction on the production of inflammatory mediators, arachidonic acid and its derivatives present in nerve injuries. With this, it promotes regeneration after damage (11,20,21). Low-level laser therapy was beneficial for this group of patients on recovery of neurosensory impairment of mandibular nerve, compared to a placebo. This was clinically determined, both in a subjective and objective way, through a neurosensory evaluation, which permitted the complete description of the neurosensory impairment evolution up to 6 months post a BSSO.

In terms of conflicts of interest, there are no financial or personal relationships with other people or organisations that could innapropiately influence this work.
